# Cardiolipin enhances the enzymatic activity of cytochrome *bd* and cytochrome *bo*_3_ solubilized in dodecyl-maltoside

**DOI:** 10.1038/s41598-021-87354-0

**Published:** 2021-04-13

**Authors:** Amer H. Asseri, Albert Godoy-Hernandez, Hojjat Ghasemi Goojani, Holger Lill, Junshi Sakamoto, Duncan G. G. McMillan, Dirk Bald

**Affiliations:** 1 Department of Molecular Cell Biology, Amsterdam Institute of Molecular and Life Sciences (AIMMS), Faculty of Science, Vrije Universiteit Amsterdam, De Boelelaan 1108, 1081 HZ Amsterdam, The Netherlands; 2 Department of Biochemistry, Faculty of Science, King Abdulaziz University, Jeddah, 21589 Saudi Arabia; 3 Department of Biotechnology, Delft University of Technology, Van der Maasweg 9, 2629 HZ Delft, The Netherlands; 4 Department of Bioscience and Bioinformatics, Kyushu Institute of Technology, Kawazu 680-4, Iizuka, Fukuoka-ken 820-8502 Japan

**Keywords:** Enzymes, Chemical biology

## Abstract

Cardiolipin (CL) is a lipid that is found in the membranes of bacteria and the inner membranes of mitochondria. CL can increase the activity of integral membrane proteins, in particular components of respiratory pathways. We here report that CL activated detergent-solubilized cytochrome *bd,* a terminal oxidase from *Escherichia coli.* CL enhanced the oxygen consumption activity ~ twofold and decreased the apparent *K*_M_ value for ubiquinol-1 as substrate from 95 µM to 35 µM. Activation by CL was also observed for cytochrome *bd* from two Gram-positive species, *Geobacillus thermodenitrificans* and *Corynebacterium glutamicum,* and for cytochrome *bo*_3_ from *E. coli*. Taken together, CL can enhance the activity of detergent-solubilized cytochrome *bd* and cytochrome *bo*_3_.

Cardiolipin (CL) is an anionic phospholipid that consists of two phosphatidyl groups connected by a glycerol moiety. CL is important for optimal function of various eukaryotic and prokaryotic membrane protein complexes^1–5^. CL can interact with bacterial respiratory complexes from phylogenetically diverse species, such as *Mycobacterium phlei*^6^, *Rhodobacter sphaeroides*^7^ and *Escherichia coli*^8^. Among *E. coli* respiratory chain complexes, CL was shown to activate purified, detergent-free cytochrome *bo*_3_^9^ and was the most efficient phospholipid for activation of detergent-solubilized NADH dehydrogenase^10^ and of liposome-reconstituted nitrate reductase^11^. Defined binding sites for CL have been determined in crystal structures of *E. coli* formate dehydrogenase N^12^, succinate dehydrogenase^13^, and nitrate reductase^11^.

The respiratory chain in *Escherichia coli* features a heme-copper-type terminal oxidase, cytochrome *bo*_3_, which transfers electrons from quinol-type substrates onto molecular oxygen. Next to this energetically efficient terminal oxidase, *E. coli* utilizes cytochrome *bd* as an alternative branch of the respiratory chain. Cytochrome *bd* oxidizes quinols, like ubiquinol or menaquinol, coupled with reduction of molecular oxygen to water (Fig. 1A)^14,15^. Cytochrome *bd* is particularly important under conditions of stress, such as O_2_-limitation^16^, in the presence of nitric oxide^17,18^ hydrogen peroxide^19–21^ and hydrogen sulfide^22,23^. Lack of cytochrome *bd* in uropathogenic *E. coli* strains led to attenuation in mouse infection models^24^.

**Figure 1:**
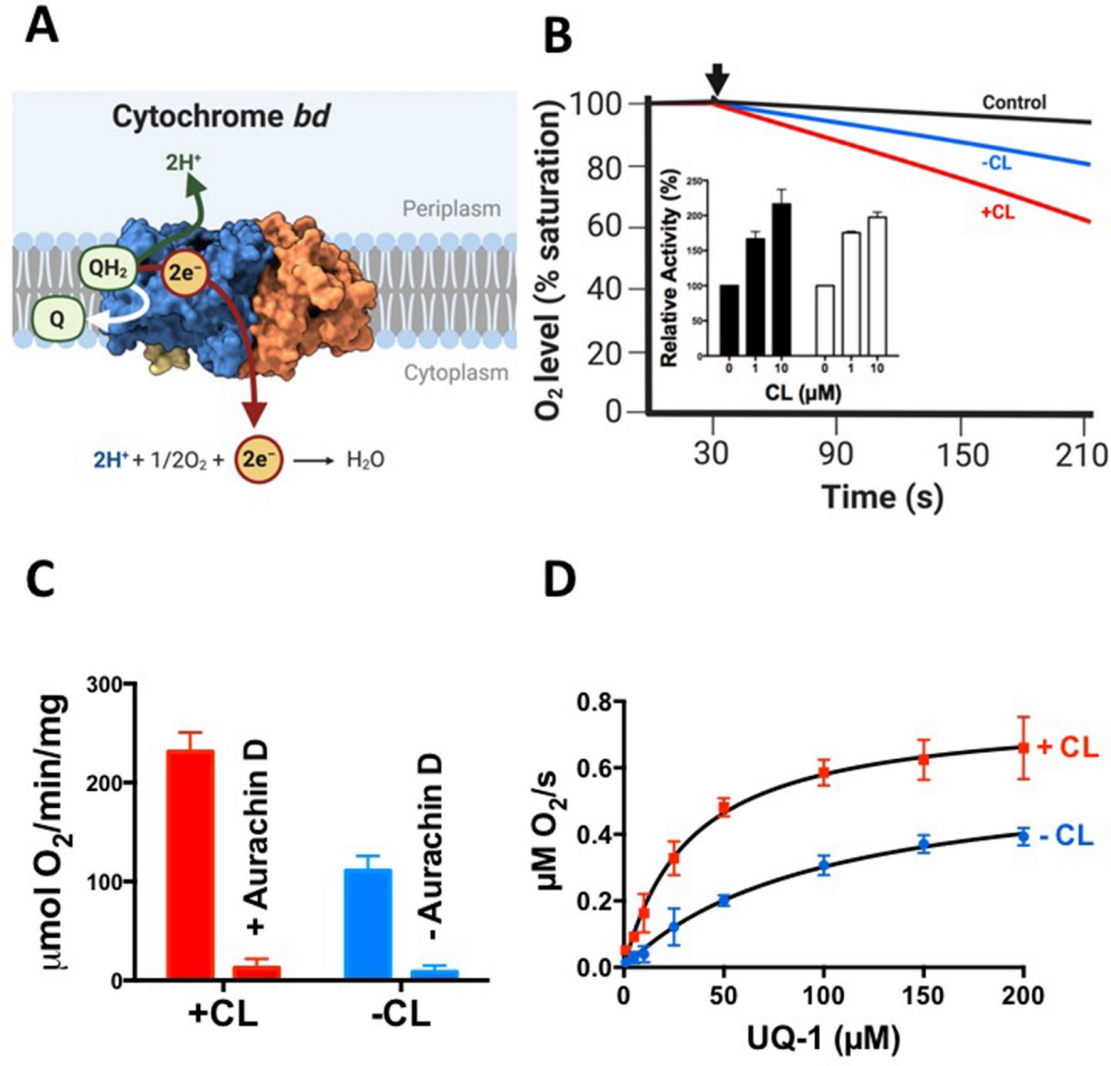
Activation of *E. coli* cytochrome *bd* by CL. (**A**) Enzymatic function of cytochrome *bd* (PDB ID: 6RKO^42^, figure created with BioRender^52^). (**B**) The effect of CL (10 μM) on oxygen consumption activity by cytochrome *bd* (final conc. 2 nM) purified from *E. coli* was determined using a Clark-type electrode. The reaction was initiated by addition of ubiquinone-1 + DTT (arrow), the negative control contained ubiquinone-1 and DTT, but no cytochrome *bd*. Inset: Dependency of activation on the pre-incubation time at the indicated CL concentrations. The enzyme was incubated with CL prior to starting the reaction for either 3 min (black bars) or 60 min (white bars). (**C**) Impact of the inhibitor aurachin D (400 nM) on oxygen consumption by cytochrome *bd* in the presence or absence of 10 µM CL. (**D**) Effect of CL on the *K*_M_ value of *E. coli* cytochrome *bd*. Curve fit was done with a simple Michaelis–Menten analysis; R^2^ values in the absence and presence of CL were 0.978 and 0.969, respectively. Average values were calculated from at least two biological replicates at 37 °C; error bars represent standard deviations.

Cytochrome *bd* is present in of a broad variety of Gram-positive and Gram-negative bacteria and archea, but not in the respiratory chain of eukaryotes^14^. Purified active cytochrome *bd* has been prepared from several bacterial species, including *E. coli*^25,26^, *Azotobacter vinelandii*^27^, *Corynebacterium glutamicum*^28^ and *Geobacillus thermodenitrificans*^29,30^. However, to our knowledge there are no data available concerning the effect of CL on cytochrome *bd* activity.

In this report, we investigated the influence of CL on detergent-purified cytochrome *bd*. We found that CL activated the enzymatic activity of cytochrome *bd* from *E. coli*, *G. thermodenitrificans* and *C. glutamicum*. We then extended our experimentation and also assessed the impact of CL on the activity of purified cytochrome *bo*_3_ from *E. coli*.

## Results

### CL enhances the activity and decreases the *K*_M_ value of purified *E. coli* cytochrome *bd*

Cytochrome *bd* was purified from *E. coli* strain MB43 using streptactin affinity chromatography and β-D-dodecyl-maltoside (DDM) as detergent, as performed earlier^32^. The purity and spectroscopic properties of the isolated protein were comparable to previous results^31,32^ (data not shown), Blue-Native PAGE showed that the sample was devoid of large-scale aggregation (Suppl. Figure 1). In line with earlier data^21,31,32^, the purified enzyme showed a specific oxygen consumption activity of ~ 110 μmol O_2_*mg^−1^*min^−1^ in buffer containing 0.025% DDM, using ubiquinol-1 as substrate. We examined the effect of CL and observed ~ twofold activation of the oxygen consumption activity (Fig. 1B). The activity of cytochrome *bd* in both the absence and the presence of CL was strongly suppressed by aurachin D (Fig. 1C), an inhibitor of *E. coli* cytochrome *bd*^33^.

We then investigated whether activation of cytochrome *bd* by CL is only observed at saturating substrate concentrations or if the *K*_M_ value changes as well. In the absence of CL, cytochrome *bd* showed a *K*_M_ value of 95 ± 16 µM for ubiquinol-1 as substrate, in line with previously published results^25,26,34^. In the presence of 10 μM CL, the *K*_M_ value decreased to 35 ± 4 μM (Fig. 1D). These results show that CL can influence enzymatic parameters of DDM-solubilized *E. coli* cytochrome *bd*.

### CL activates purified cytochrome *bd* from Gram-positive bacteria

Next, we evaluated if activation by CL can also be found for cytochrome *bd* purified from other bacteria. Genetic classification analyses indicated that two basic types of cytochrome *bd* can be distinguished, based on the length of a hydrophilic loop (Q-loop) close to the substrate binding site^14,35–37^. Whereas *E. coli* cytochrome *bd* displays a long Q-loop, cytochrome *bd* from Gram-positive bacteria harbors a short version^14,35–37^. Previously, purification of cytochrome *bd* from the two Gram-positive strains *Geobacillus thermodenitrificans* (formerly called *Bacillus stearothermophilus*)^29,30^ and *Corynebacterium glutamicum*^28^ was described. As observed above for the *E. coli* enzyme, purity and spectroscopic properties of these isolated proteins were comparable to previous results^28–30^ (data not shown) and the samples were devoid of large-scale aggregation (Suppl. Figure 1).

**Figure 2:**
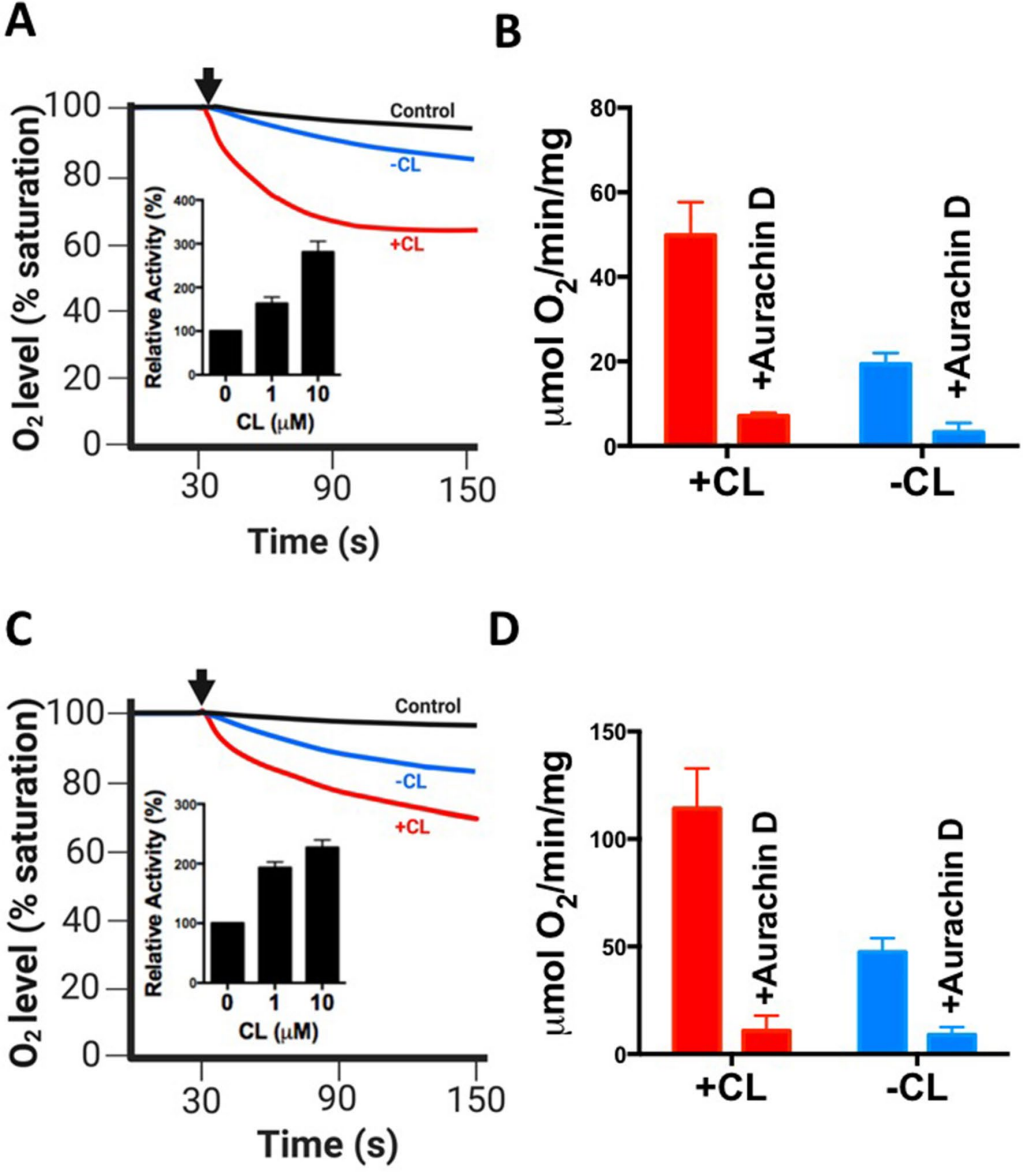
Activation of cytochrome *bd* from Gram-positive bacteria by CL. The effect of CL (final conc. 10 μM) on oxygen consumption activity of cytochrome *bd* purified from *G. thermodenitrificans* (final conc. 10 nM) (**A,B**) and *C. glutamicum* (final conc. 2.8 nM) (**C,D**) was determined using a Clark-type electrode at 37 °C. The reaction was initiated by addition of menaquinone-1 + DTT (arrow), the negative control contained menaquinone-1 and DTT, but no cytochrome *bd*. Insets (**A,C**) Dependency of activation on the CL concentration. (**B,D**) Impact of the inhibitor aurachin D (400 nM) on the oxygen consumption activity in the presence or absence of 10 µM CL. Average values were calculated from at least two biological replicates; error bars represent standard deviations.

We examined the oxygen consumption activity of purified cytochrome *bd* from both strains with the same protocol as for *E. coli* cytochrome *bd*, except for using menaquinol-1 instead of ubiquinol-1 as substrate, as these Gram-positive bacteria use menaquinone as main constituent of the quinone pool. Cytochrome *bd* from *G. thermodenitrificans* showed lower oxygen consumption activity (~ 18 μmol O_2_*mg^−1^*min^−1^ in the initial phase) as compared to the *E. coli* enzyme, consistent with previous data^38^. After the initial phase of the reaction, time-dependent inactivation was observed (Fig. 2A). CL significantly increased the activity of cytochrome *bd* from this strain (Fig. 2A). As observed above for the *E. coli* enzyme, the activity of *G. thermodenitrificans* cytochrome *bd* was sensitive to inhibition by aurachin D in the presence and absence of CL (Fig. 2B).

Consistent with previous results^28^, the oxygen consumption activity of cytochrome *bd* from *C. glutamicum* (~ 50 μmol O_2_*mg^1^*min^−1^) was lower than that of the *E. coli* enzyme, but higher than that of *G. thermodenitrificans* cytochrome *bd*. Importantly, the activity was significantly enhanced by CL (Fig. 2C). We confirmed that the observed oxygen consumption activity in the presence and absence of CL was sensitive to inhibition by aurachin D (Fig. 2D). These results reveal that activation by CL is not restricted to cytochrome *bd* from *E. coli*, but can also be found for this enzyme isolated from two Gram-positive bacteria.

### CL activates enzymatic activity of cytochrome *bo*_3_ from *E. coli*

**Figure 3:**
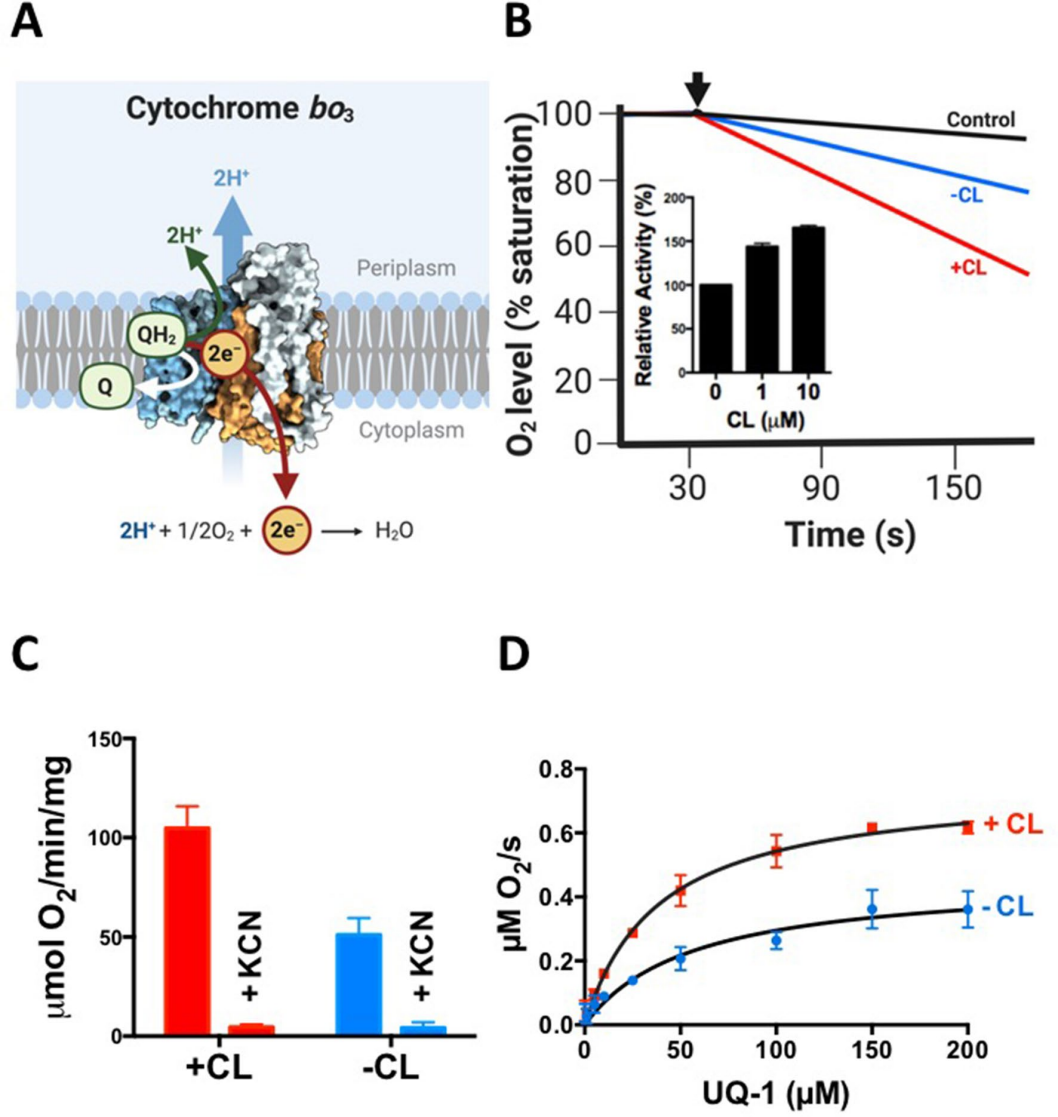
Activation of *E. coli* cytochrome *bo*_3_ by CL. (**A**) Enzymatic function of cytochrome *bo*_3_ (PDB ID: 1FFT^39^, figure created with BioRender^52^) (**B**) The effect of CL (final conc. 10 μM) on oxygen consumption activity by cytochrome *bo*_3_ purified from *E. coli* (final conc. 5 nM) was determined using a Clark-type electrode. The reaction was initiated by addition of ubiquinone-1 + DTT (arrow), the negative control contained ubiquinone-1 and DTT, but no cytochrome *bo*_3_. Inset: dependency of activation on the CL concentration. (**C**) Impact of KCN (2 mM) on oxygen consumption by cytochrome *bo*_3_ in the presence or absence of 10 µM CL. (**D**) Effect of CL on the *K*_M_ value of purified *E. coli* cytochrome *bo*_3_. Curve fit was done with a simple Michaelis–Menten analysis, R^2^ values in the absence and presence of CL were 0.974 and 0.983, respectively. All experiments were carried out at 37 °C. Average values were calculated from at least two biological replicates; error bars represent standard deviations.

We then extended our efforts to the second terminal oxidase found in *E. coli*, cytochrome *bo*_3_. Cytochrome *bo*_3_ is a heme-copper-type quinol oxidase and evolutionary is not related to cytochrome *bd*^14,39^. Cytochrome *bo*_3_ was purified from *E. coli* strain GO105/pJRhisA using DDM as detergent without significant aggregation (Suppl. Figure 1), displaying similar spectroscopic properties as described earlier^40,41^ (data not shown). Like cytochrome *bd*, cytochrome *bo*_3_ can accept ubiquinol-1 as electron donor and reduces molecular oxygen (Fig. 3A). In the absence of CL, cytochrome *bo*_3_ displayed a specific oxygen consumption activity of 47 μmol O_2_*mg^−1^*min^−1^, comparable to previously reported values^39,40^. Addition of CL caused a pronounced increase in activity (Fig. 3B). Oxygen consumption by cytochrome *bo*_3_ in both the absence and in the presence of CL was highly susceptible to the inhibitor potassium cyanide (KCN) (Fig. 3C). The *K*_M_ value decreased from 56 ± 13 μM in the absence of CL to 38 ± 4 μM in the presence of CL (Fig. 3D). Previously, a *K*_M_ of 59 μM has been reported for cytochrome *bo*_3_ in the presence of CL in the detergent-free state^8^. Taken together, our results show that CL can activate both terminal oxidases in *E. coli*.

## Discussion

It has been established that CL can enhance the activity of various bacterial membrane proteins, including complexes of both aerobic and of anaerobic respiration^1,2,5,9,11,12^. Previously, activation of purified cytochrome *bo*_3_ by CL and activation of purified cytochrome *bd* by asolectin was reported^9,26^. However, these experiments were carried out in detergent-free state. In the absence of detergent, membrane protein aggregation likely causes a significant decrease in activity, which subsequently is relieved by addition of lipid. In this study, we found that CL enhanced the activity of both terminal oxidases of the *E. coli* respiratory chain in the detergent-solubilized state. In line with our results, recently high enzymatic activity (889 e^−^*s^−1^ ≅ 135 μmol O_2_*mg^−1^*min^−1^) has been reported for *E. coli* cytochrome *bd* solubilized in MSP1D1/POPC-containing nano-discs^42^, likely reflecting the importance of the lipid environment for the performance of this enzyme.

CL can be located at the outer surface of a detergent-solubilized membrane protein, enabling proper vertical positioning of the protein, or it may bind to clefts or cavities on the protein surface^1,3^. CL may play a structural role, e.g. by binding at the interface between individual subunits, as previously reported for formate dehydrogenase N^12^. Alternatively, CL may enhance the interaction with the quinol substrate and/or facilitate the electron transfer reaction, as reported for nitrate reductase, where CL binds to a niche near the quinol-binding site^11^. In the respiratory chain of *Saccharomyces cerevisiae* CL stabilizes the super-complex formed by the cytochrome *bc*_1_ complex and cytochrome *c* oxidase, binding at the interface of the two components^5,43^. In case of mitochondrial ATP synthase, CL transiently binds to conserved lysine residues in subunit c, possibly lubricating the motion of this membrane-embedded rotary machine^44^. As found for DDM-purified cytochrome *c* oxidase from bovine heart mitochondria, CL can be functionally required for optimal electron transports and proton translocation^43,44^. Three-dimensional structures are available for cytochrome *bd* from *Geobacillus thermodenitrificans*^30^ and from *E. coli*^42,45^, however, the presently achieved resolution might not allow for identification of all bound lipid molecules. The decreased *K*_M_ value of *E. coli* cytochrome *bd* for ubiquinol-1 measured here indicates that CL influences the substrate binding process.

In our study we investigated cytochrome *bd* and cytochrome *bo*_3_ in the detergent-solubilized state and our results therefore do not clarify if CL has a similar effect on these enzymes in the native membrane. CL as high-curvature lipid is predominantly localized at the poles in rod-shaped bacteria and may thereby influence the cellular localization of membrane protein complexes^46^, as suggested for the SecYEG translocon^47^. Previously, for cytochrome *bd*, a distribution in mobile patches in the *E. coli* cytoplasmic membrane has been reported^48^. It needs to be investigated if CL can influence function, localization or dynamics of cytochrome *bd* or cytochrome *bo*_3_ in the native plasma membrane.

## Materials and methods

### Chemicals

Aurachin D was synthesized as described earlier in Li et al. 2013^49^ and was kindly provided by Dr. Jennifer Herrmann (Helmholtz Centre for Infection Research and Pharmaceutical Biotechnology, Saarbrücken). CL was purchased from Sigma (C1649, from bovine heart, > 80% polyunsaturated fatty acid content, primarily linoleic acid). All other chemicals were bought from Sigma, unless indicated otherwise.

### Purification of cytochrome *bd*

Cytochrome *bd* from *E. coli* was purified based on Hoeser et al.^31^, with modifications as described by Goojani et al.^32^. Briefly, *E. coli* MB43 carrying the pET17cydABX-Strep-tag plasmid was grown in Luria–Bertani (LB) medium with 100 µg/ml Ampicillin at 37 °C overnight with shaking at 200 rpm. The bacteria were diluted to OD_600_ ~ 0.01 in 800 ml LB medium with 100 µg/ml Ampicillin and incubated until reaching OD_60_ ~ 0.4. Then IPTG (0.45 mM final conc.) was added and the bacteria were incubated again at 37 °C, 200 rpm until reaching OD_600_ ~ 2.0. Cells were sedimented by centrifugation at 6000 g for 20 min (JA-10 rotor). The pellets were washed by phosphate buffer saline, pH 7.4, and spun down at 6000 g for 20 min. Each 15 g of wet cells were re-suspended with 75 ml of MOPS solution (50 mM 3-N-morpholino-propanesulfonic acid, 100 mM NaCl and protease inhibitor (cOmplete, Roche). The cells were disrupted by passing three times though a Stansted cell homogenizer at 1.8 kb. Unbroken cells were centrifuged at 9500 g (Ja-3050-ti rotor) for 20 min. Subsequently, the supernatant was pelleted by ultracentrifugation 250,000 g (70-ti rotor) for 75 min at 4 °C. The pellet was re-suspended in MOPS solution and the protein concentration was measured using the BCA Protein Assay kit (Pierce) as described by the manufacturer. The concentration was adjusted to 10 mg/ml and incubated in MOPS solution containing 1% DDM (final conc.) at 4 °C for an hour with gentle shaking. Un-solubilized material was sedimented by ultracentrifugation at 250,000 g at 4 °C for 15 min (70-ti rotor). The collected supernatant was applied on streptactin column at 4 °C (cold room) and the flowthrough was collected. The column was washed with washing buffer (50 mM sodium phosphate, 300 mM NaCl, protease inhibitor (cOmplete), containing 0.01% DDM, pH 8.0) to remove unspecific protein binding and the flow-through was collected again. The elution buffer (50 mM sodium phosphate, 300 mM NaCl, protease inhibitor (C0mplete EDTA free), 0.01% DDM, and 2.5 mM desthiobiotin pH 8.0) was added to the column at 4 °C to elute the protein.

### Purification of cytochrome *bd* from* Geobacillus thermodenitrificans* and from* Corynebacterium glutamicum*

Cytochrome *bd* from *G. thermodenitrificans* was extracted and purified from membrane fractions of *G. thermodenitrificans* K1041/pSTE-*cbdAB* recombinant cells with two consecutive column chromatography of DEAE-Toyopearl and hydroxyapatite in the presence of 0.5% (w/v) MEGA9 + 10, as described previously in Arutyunyan et al. 2012^36^. Cytochrome *bd* from *C. glutamicum* was extracted and purified from membrane fractions of *C. glutamicum* Δ*ctaD*/pPC4-*cydABDC* recombinant cells^50^ with two consecutive chromatography of hydroxyapatite and then DEAE-Toyopearl in the presence of 0.05% (w/v) DDM.

### Purification of* E. coli* cytochrome* bo*_3_

Cytochrome *bo*_3_ was extracted and purified from *E. coli* cytoplasmic membranes based on Rumbley et al. 1997^40^, with modifications as described in Hards et al. 2018^41^. *E. coli* cytoplasmic membranes were prepared from strain GO105/pJRhisA in which cytochrome *bo*_3_ is overexpressed. *E. coli* was aerobically grown to mid-log phase at 37 °C in LB medium supplemented with 500 μM CuSO_4_ and 100 μg ml^−1^ carbenicillin. Cells were harvested by centrifugation at 10,000 × *g* for 10 min and the pellets were washed and repelleted twice with buffer A (20 mM (3-N-morpholino-propanesulfonic acid (MOPS), 30 mM Na_2_SO_4_, pH 7.4). Cells were then resuspended in buffer A containing a mini protease inhibitor tablet (cOmplete) per 50 mL, 0.1 mM phenylmethylsulfonyl fluoride, 0.1 mg/ml pancreatic DNase, and lysed by two passages through a French Press at 20,000 psi. Any debris and unbroken cells were removed by centrifugation at 10,000 × *g* for 30 min. The supernatant was then ultracentrifuged (200,000 × *g*, 45 min, 4 °C) and the membrane pellet resuspended in buffer B (20 mM MOPS, 30 mM Na_2_SO_4_, 25% w/w sucrose, pH 7.4). The suspension was applied to the top of a 30% w/w to 55% w/w sucrose gradient and ultracentrifugation (130,000 × *g*, 16 h, 4 °C) with no deceleration or breaking to separate inner membrane from outer membrane. The inner membrane fraction was removed from the sucrose gradient and washed 3 times with buffer A by ultracentrifugation (200,000 × *g*, 45 min, 4 °C). Inner membranes were then resuspended in buffer A and either used immediately for purification or stored in aliquots at − 80 °C until use. To extract cytochrome *bo*_3_, inner membrane fractions were diluted to 5 mg/mL protein content with solubilization buffer (20 mM Tris HCl, pH 8.0, 5 mM MgSO_4_, 10% glycerol, 300 mM NaCl, 1% DDM, 10 mM imidazole) and incubated at 30 °C for 30 min with gentle inversion every 5 min. The unsolubilized material was removed by ultracentrifugation (200,000 × *g*, 45 min, 4 °C), and the supernatant was applied to a Nickel-Sepharose High Performance (GE Healthcare) column that was previously washed with water and equilibrated with IMAC buffer (50 mM Tris/HCl, pH 8.0, 5 mM MgSO_4_, 10% glycerol, 0.01% DDM, 300 mM NaCl) containing 10 mM imidazole. To remove contaminating proteins, the resin was washed with IMAC buffer containing 30 mM imidazole and 150 mM NaCl, and cytochrome *bo*_3_ was eluted with IMAC buffer containing with 200 mM imidazole, 150 mM NaCl, and 20% glycerol. The red cytochrome *bo*_3_ containing fractions were pooled and concentrated to 6.57 mg mL^−1^ using an Amicon Ultra centrifugal filter devices with100,000 Da molecular weight cutoff.

### Oxygen consumption activity assay

Oxygen consumption by purified cytochrome *bd* and cytochrome *bo*_3_ was measured using a Clark-type electrode as previously described in Lu et al.^51^, with modifications as in Goojani et al.^31^. Briefly, the electrode was fully aerated (212 μM O_2_ at 37 °C) and calibrated with sodium hydrosulfite. The purified enzymes (final conc: 2 nM for cytochrome *bd* from *E. coli*, 10 nM for cytochrome *bd* from *G. thermodenitricifans*, 2.8 nM for cytochrome *bd* from *C. glutamicum*, 5 nM for cytochrome *bo*_3_) were pre-incubated for three minutes with CL (and with inhibitors, if applicable) in a pre-warmed (37 °C) buffer containing 50 mM 3-N-morpholino-propanesulfonic acid (MOPS), 100 mM NaCl and 0.025% DDM, pH 7.5. Ubiquinone-1 (Sigma) and menaquinone-1 (Santa Cruz Biotechnology) were dissolved in absolute ethanol (20 mM stock) and the reducing agent dithiothreitol (1 M stock) in 50 mM HEPES (4- (2-hydroxyethyl) piperazine-1-ethanesulfonic acid), pH 7.75. Quinone stock and DTT stock were mixed in 1:1 volume ratio and incubated for 3 min (ubiquinone-1/DTT) or 6 min (menaquinone-1/DTT) at 37 °C. The oxygen consumption reaction was initiated by adding the quinone/DTT mixture (final concentration 200 μM quinone and 10 mM DTT) to the assay mixture, respiration was measured for 3 min. The enzymatic activity was calculated from the slope in the period 30 s—60 s after starting the reaction (linear approximation).

## Supplementary Information

Supplementary Information 1.

Supplementary Information 2.

## Data Availability

The original data describing rates measured in this study are compiled in a supplementary file (Suppl. Table 1). Original time courses generated during the current study are available from the corresponding author on reasonable request.
