# Supplementary material for: Cardiolipin enhances the enzymatic activity of cytochrome bd and cytochrome bo3 solubilized in dodecyl-maltoside
Source: Sci Rep. 2021 Apr 13;11:8006. doi: 10.1038/s41598-021-87354-0 (PMC8044227; doi:10.1038/s41598-021-87354-0)
Supplement: Supplementary file 1 — Supplementary Information 1. [file 41598_2021_87354_MOESM1_ESM.docx]

**Supplementary Information**

**Cardiolipin enhances the enzymatic activity of**

**cytochrome *bd* and cytochrome *bo*_3_ solubilized**

**in dodecyl-maltoside**

Amer H. Asseri^1,2#^, Albert Godoy-Hernandez^3#^, Hojjat Ghasemi Goojani^1^, Holger Lill^1^, Junshi Sakamoto^4^, Duncan McMillan^3^* & Dirk Bald^1^*

^1^ Department of Molecular Cell Biology, Amsterdam Institute of Molecular & Life Sciences, (AIMMS), Faculty of Science, Vrije Universiteit Amsterdam, De Boelelaan 1108, 1081 HZ, Amsterdam, The Netherlands.

^2^ Department of Biochemistry, Faculty of Science, King Abdulaziz University, Jeddah, 21589, Saudi Arabia.

^3^ Department of Biotechnology, Delft University of Technology, Van der Maasweg 9, 2629 HZ Delft, The Netherlands

^4^ Department of Bioscience & Bioinformatics, Kyushu Institute of Technology, Kawazu 680-4, Iizuka, Fukuoka-ken, Japan.

# The authors contributed equally.

* To whom correspondence should be addressed: [d.bald@vu.nl](mailto:d.bald@vu.nl) or D.G.G.McMillan@tudelft.nl.

**Supplementary Figure 1**

Blue-Native PAGE analysis of purified cytochrome *bd* from *E. coli* (A), cytochrome *bd* from *C. glutamicum* (B), cytochrome *bd* from *G. thermodenitrificans* (C), and cytochrome *bo*_3_ from *E. coli* (D). 3-12% Bis-Tris Mini-gels (Novex Life Technologies) were used according to the manufacturer’s instructions.
